# Care of Patients with Male Hypogonadism: A Joint Position Statement from the Brazilian Society of Endocrinology and Metabolism (SBEM), the Brazilian Society of Urology (SBU), and the Brazilian Association for Sexual Medicine and Health (ABEMSS)

**DOI:** 10.1590/S1677-5538.IBJU.2025.0610

**Published:** 2026-02-05

**Authors:** Alexandre Hohl, Leonardo Lopes, Marcelo Fernando Ronsoni, Eduardo P. Miranda, Tayane Muniz Fighera, Fernando Nestor Facio, Lucas Bandeira Marchesan, Luiz Otavio Torres

**Affiliations:** 1 Universidade Federal de Santa Catarina Florianópolis SC Brasil Serviço de Endocrinologia e Metabologia, Universidade Federal de Santa Catarina – UFSC, Florianópolis, SC, Brasil; 2 Disciplina de Urologia Faculdade de Medicina do ABC Santo André SP Brasil Disciplina de Urologia Faculdade de Medicina do ABC, Santo André, SP, Brasil; 3 Universidade Federal de Santa Catarina Departamento Clínica Médica Florianópolis SC Brasil Departamento Clínica Médica, Universidade Federal de Santa Catarina – UFSC, Florianópolis, SC, Brasil; 4 Centro Universitario Christus Fortaleza CE Brasil Centro Universitario Christus, Fortaleza, CE, Brasil; 5 Universidade Federal do Rio Grande do Sul Departamento de Medicina Interna Porto Alegre RS Brasil Departamento de Medicina Interna, Universidade Federal do Rio Grande do Sul – UFRS, Porto Alegre, RS, Brasil; 6 Faculdade de Medicina de São José do Rio Preto São José do Rio Preto SP Brasil Disciplina de Urologia, Faculdade de Medicina de São José do Rio Preto - FAMERP, São José do Rio Preto, SP, Brasil; 7 Hospital Nossa Senhora da Conceição Porto Alegre RS Brasil Serviço de Endocrinologia e Metabologia, Hospital Nossa Senhora da Conceição, Porto Alegre, RS, Brasil; 8 Centro Universitário de Belo Horizonte Campus Antonio Carlos Belo Horizonte Serviço de Urologia MG Brasil Serviço de Urologia, Centro Universitário de Belo Horizonte Campus Antonio Carlos Belo Horizonte, MG, Brasil

**Keywords:** Hypogonadism, Testosterone, Infertility, Male

## Abstract

Male hypogonadism is a prevalent and clinically relevant condition with substantial effects on reproductive, metabolic, skeletal, and psychosocial health. Rising obesity rates, metabolic syndrome, and anabolic-androgenic steroid use have increased the frequency of functional hypogonadism in Brazil. Despite advances in diagnosis and treatment, clinical practice remains heterogeneous and access to standardized recommendations is limited.

This joint position statement from the Department of Female Endocrinology, Andrology and Transgenderism (DEFAT) of the Brazilian Society of Endocrinology and Metabolism (SBEM), the Brazilian Society of Urology (SBU), and the Brazilian Association for Sexual Medicine and Health (ABEMSS) provides practical, evidence-based guidance for the evaluation and management of male hypogonadism in Brazil. The document outlines diagnostic criteria, including morning total testosterone confirmation and assessment of gonadotropins, and emphasizes recognition of functional etiologies such as obesity-related hypogonadism. Therapeutic recommendations include testosterone replacement therapy for confirmed organic hypogonadism, preferential use of long-acting intramuscular or transdermal formulations, and fertility-preserving strategies (SERMs, hCG, aromatase inhibitors) when indicated. The statement also addresses monitoring protocols, safety considerations, and the management of adverse effects.

This is the first multidisciplinary Brazilian guideline harmonizing endocrine, urological, and sexual medicine perspectives to support national clinical practice. This consensus aims to promote consistent clinical decision-making, reduce underdiagnosis and overtreatment, and ensure safe, individualized care aligned with international principles and adapted to the national context.

## INTRODUCTION

Male hypogonadism is a common endocrine disorder with significant clinical, metabolic, and psychosocial consequences. Its recognition and management have become increasingly complex due to the rising prevalence of obesity, metabolic syndrome, and the recreational or therapeutic use of anabolic-androgenic steroids (AAS). These factors not only affect circulating testosterone levels but also exert far-reaching effects on fertility, bone health, and overall quality of life.

Advances in understanding the pathophysiology of hypogonadism have emphasized the need for individualized, evidence-based therapeutic strategies. Testosterone replacement therapy remains the primary treatment for most patients with male hypogonadism. Selective estrogen receptor modulators are effective alternatives, particularly for men with functional hypogonadism who wish to preserve fertility. Current consensus guidelines advocate a multidisciplinary approach integrating endocrinology, urology, sexual medicine, and primary care to achieve optimal patient-centered outcomes.

This position statement introduces the first multidisciplinary Brazilian consensus developed jointly by the Brazilian Society of Endocrinology and Metabolism (SBEM), the Brazilian Society of Urology (SBU), and the Brazilian Association for Sexual Medicine and Health (ABEMSS) to guide the diagnosis and management of male hypogonadism; assist in the standardization of diagnostic thresholds and therapeutic targets for testosterone replacement in the Brazilian context and offers the adaptation of international evidence to local clinical practice and regulatory availability.

This position statement applies evidence-graded recommendations based on the strength and quality of available data, adapted from internationally recognized endocrine and urological guideline frameworks. Levels of evidence reflect methodological rigor and consistency of supporting studies, and recommendations are categorized according to clinical benefit and risk. When high-quality evidence is limited, expert consensus from specialists in endocrinology, urology, and sexual medicine was applied to guide best practice in the Brazilian setting. This approach ensures scientific rigor while maintaining practical applicability in routine clinical care.

## DIAGNOSIS OF HYPOGONADISM IN MEN

### Definition and Classification

Male hypogonadism is defined as a clinical syndrome characterized by a combination of specific signs and symptoms and confirmed biochemical evidence of testosterone deficiency. It reflects impaired testicular function resulting in decreased production of androgens and/or impaired spermatogenesis, with potential adverse effects on multiple organ systems and quality of life (1–5).

Hypogonadism is broadly classified into primary (hypergonadotropic) and secondary (hypogonadotropic) forms. Primary hypogonadism arises from intrinsic testicular failure, typically accompanied by elevated gonadotropin levels (2, 3). Secondary hypogonadism results from impaired hypothalamic or pituitary stimulation of the testes, characterized by low or inappropriately normal levels of gonadotropins (2–4). A compensated form is also recognized, characterized by normal testosterone levels with elevated luteinizing hormone (LH) (3). A third category, androgen resistance, refers to rare conditions where tissue insensitivity to testosterone is present, despite normal or elevated circulating testosterone levels. This includes partial or complete androgen insensitivity syndrome and enzymatic defects affecting androgen metabolism (3).

An important emerging concept is functional hypogonadism, which affects both young, middle-aged and older men. Functional hypogonadism is diagnosed when no identifiable organic pathology of the hypothalamic-pituitary-gonadal (HPG) axis is present. It is frequently associated with chronic systemic diseases, obesity, diabetes mellitus, and inflammatory conditions, all of which can impair the HPG axis and testosterone production (2, 3). Functional hypogonadism is often reversible with resolution or optimization of the underlying condition.

### Physiology of the Hypothalamic-Pituitary-Gonadal (HPG) Axis

**Figure 1:**
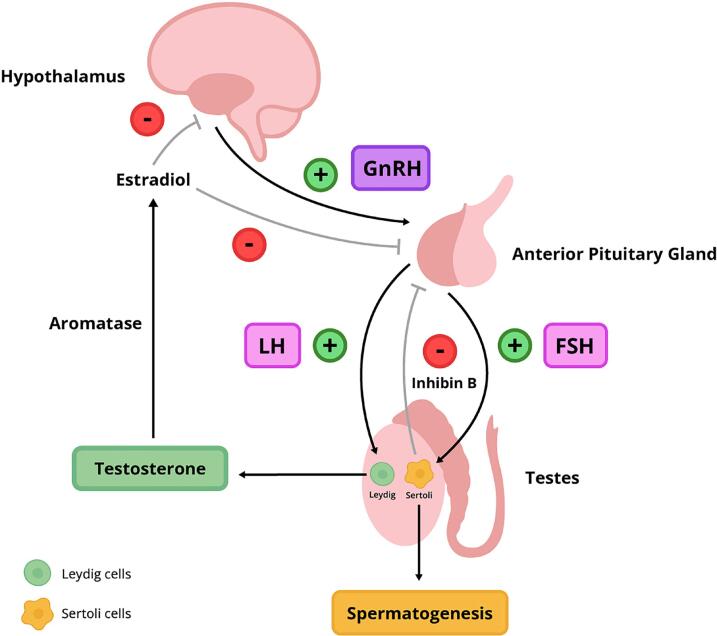
Schematic representation of the hypothalamic–pituitary–gonadal (HPG) axis and its regulatory feedback mechanisms.

Clinicians must recognize that the HPG axis is fundamental for the regulation of male reproductive function and testosterone homeostasis. The hypothalamus secretes gonadotropin-releasing hormone (GnRH) in a pulsatile fashion, which stimulates the anterior pituitary to release LH and follicle-stimulating hormone (FSH) (4, 6). LH primarily stimulates Leydig cells in the testes to produce testosterone, whereas FSH acts on Sertoli cells to promote spermatogenesis and inhibin B production. It is essential to maintain the physiological pulsatility of GnRH, as continuous stimulation can lead to receptor desensitization and impaired gonadotropin secretion (7). Figure-1 schematically describes the physiology of the HPG (Figure-1).

The figure illustrates the hypothalamic–pituitary–gonadal (HPG) axis and the endocrine feedback loops that regulate male reproductive function. Gonadotropin-releasing hormone (GnRH) is secreted by the hypothalamus and stimulates the anterior pituitary to release luteinizing hormone (LH) and follicle-stimulating hormone (FSH). LH acts on Leydig cells to promote testosterone synthesis, whereas FSH acts on Sertoli cells to support spermatogenesis and stimulate the production of inhibin B. Testosterone and estradiol (via aromatization) exert negative feedback at both the hypothalamic and pituitary levels, while inhibin B selectively suppresses pituitary FSH secretion. The coordinated action of these hormones maintains testicular function and regulates male reproductive homeostasis.

GnRH = gonadotropin-releasing hormone; LH = luteinizing hormone; FSH = follicle-stimulating hormone; HPG = hypothalamic–pituitary–gonadal.

Healthcare providers should be aware that testosterone exerts widespread effects across multiple organ systems, mediated through androgen receptors. Approximately 60% of circulating testosterone is bound to sex hormone-binding globulin (SHBG), 38% to albumin, and about 2% remains free and biologically active (1, 4). Peripheral conversion of testosterone to dihydrotestosterone (DHT) via 5α-reductase and to estradiol via aromatase must be considered, given their critical roles in androgenic activity and bone health, respectively. Both testosterone and estradiol exert negative feedback at the hypothalamic and pituitary levels, regulating GnRH, LH, and FSH secretion to maintain hormonal equilibrium (8).

When evaluating patients, clinicians must assess for potential disruptions at all levels of the HPG axis (9). A clear understanding of HPG axis physiology is indispensable for the precise diagnosis and individualized treatment of male hypogonadism.

**Recommendation 01:** Clinicians should perform a structured evaluation of the HPG axis, considering primary, secondary, and functional causes of hypogonadism (Class: I, Level of Evidence: B).

### Epidemiology and Prevalence in Brazil

Hypogonadism often remains underdiagnosed, underreported, and paradoxically, sometimes overtreated. Prevalence estimates vary from 2% to over 30%, depending on the population studied (10).

Understanding the epidemiology of male hypogonadism allows for the determination of the merits of androgen deficiency screening, the predictive value of diagnostic tests, and the socio-economic impact associated with the disorder (10). In the United States, approximately 481,000 new cases of hypogonadism occur annually among men aged 40 to 69 years (11).

In Brazil, the epidemiological characterization of male hypogonadism remains limited, mainly due to the lack of large-scale population studies. Nevertheless, factors such as population aging, and the high prevalence of obesity and diabetes indicate that hypogonadism represents a significant public health issue (12). The association between obesity and functional male hypogonadism (MOSH: Male Obesity Secondary Hypogonadism) is widely recognized and corroborated by international guidelines, such as those from the medical societies of Endocrinology and Metabolism and Urology (3, 12, 13).

Demographic projections indicate that the proportion of individuals aged 65 and older will increase significantly in the coming decades. The literature shows that with aging, total serum testosterone decreases by 1.6% per year, and sex hormone-binding globulin (SHBG) levels increase by 1.3% annually (2–4). These physiological changes are related to overall health status. In Brazil, essential challenges persist, including the need for representative population studies, laboratory standardization, and awareness campaigns to reduce underdiagnosis of the condition.

### Clinical Signs and Symptoms

The clinical manifestations of testosterone deficiency are predominantly nonspecific. The presentation varies according to the age at onset, severity of deficiency, presence of comorbidities, individual differences in androgen sensitivity, and prior exposure to testosterone therapy (3–5, 14). There are no population-based studies comprehensively evaluating the spectrum of symptoms and signs across the full severity range of male hypogonadism (4). Table-1 comprises specific and non-specific signs and symptoms suggestive of testosterone deficiency.

**Table 1:** Symptoms and signs suggestive of testosterone deficiency in Men (1–5, 14–19).

Specific testosterone deficiency
Incomplete or delayed sexual development
Loss of body (facial, axillary and pubic) hair
Very small testes (<6 mL)
Suggest testosterone deficiency
Decreased libido, diminished sexual thoughts
Erectile dysfunction, fewer spontaneous (morning and nocturnal) erections
Delayed ejaculation, reduced ejaculate volume, diminished orgasmic intensity
Gynecomastia
Eunuchoid body proportions
Infertility
Vasomotor symptoms (e.g., hot flushes, sweating),
Fine wrinkling of the skin (especially peri orally)
Height loss, low-trauma fractures, and reduced bone mineral density.
Nonspecific but associated with testosterone deficiency
Decreased energy levels, low or depressed mood, irritability, reduced motivation,
Fatigue
Cognitive changes (including impaired concentration and memory)
Sleep disturbances, excessive daytime sleepiness
Diminished muscle strength, reduced physical performance or activity
Increased adiposity (particularly visceral fat)
Unexplained mild normochromic, normocytic anemia

While screening questionnaires may offer clinical utility, they lack sufficient specificity to support their use in systematic screening for hypogonadism (3, 20).

In cases of suspected or confirmed hypogonadotropic hypogonadism, all individuals should be systematically queried regarding their sense of smell to evaluate for hyposmia or anosmia, which may indicate underlying genetic syndromes such as Kallmann syndrome (2–4).

**Recommendation 02:** All men should be asked during medical consultations about possible signs and clinical symptoms associated with low testosterone levels (Class: I, Level of Evidence: A).

**Recommendation 03:** We recommend against routine screening of men in the general population for hypogonadism. (Class: III, Level of Evidence: A).

**Recommendation 04:** Individuals with hypogonadism, physical examination is an essential component of clinical assessment. It should include evaluation of secondary sexual characteristics (such as facial and body hair, and pubertal development), breast tissue (gynecomastia), body composition (weight and fat distribution), muscle mass, and skeletal structure/function. Examination of the testes should assess size, consistency, and location, while penile evaluation should include length and the position of the urethral meatus. Attention should also be given to the identification of syndromic features suggestive of underlying genetic or developmental disorders (Class: I, Level of Evidence: A).

### Laboratory evaluation

The diagnosis of male hypogonadism depends on both clinical and biochemical findings. However, since clinical decisions can be made based on laboratory results at both the time of diagnosis and during follow-up, ensuring the reliability of these data is crucial. Total testosterone (TT) concentration is the recommended test for the initial evaluation of male hypogonadism. Its levels fluctuate throughout the day, peaking in the morning, and can be influenced by food and glucose intake. Therefore, blood samples should be collected in the morning, between 7 and 10 a.m., following an overnight fast. Any reduced levels should be confirmed by a second sample under the same conditions, ideally with a 4-week interval. Blood collections should not be performed during acute illness or during an exacerbation of chronic condition (4, 14, 21–24).

Conditions that affect SHBG levels can influence testosterone results. Obesity, diabetes mellitus, glucocorticoid use, androgenic and progestogenic steroids, nephrotic syndrome, acromegaly, and hypothyroidism are among the conditions that can reduce SHBG levels, thereby increasing free testosterone (FT) concentrations. Elevated SHBG levels may be associated with aging, liver disease, hyperthyroidism, HIV infection, estrogens and anticonvulsants (3, 4, 25).

In Brazil, most laboratories use automated immunoassays (chemiluminescence-based) for measuring total testosterone. Immunoassays are considered acceptable for clinical evaluation in adult men. The gold standard method for total testosterone measurement is liquid chromatography-tandem mass spectrometry (LC-MS/MS), which is not routinely available in clinical practice in Brazil. This method offers superior accuracy and specificity, particularly at low testosterone concentrations (such as in children or men with severe hypogonadism). The Endocrine Society, the International Federation of Clinical Chemistry and Laboratory Medicine (IFCC), and the CDC Hormone Standardization Program (HoSt) recommend the use of LC-MS/MS or immunoassays calibrated with traceable reference standards (4, 14).

Laboratory values for the diagnosis of hypogonadism vary across different guidelines, based on studies conducted with populations of young men without comorbidities, using LC-MS/MS as a methodology for measuring total testosterone.

Several international societies provide reference thresholds for the biochemical diagnosis of male hypogonadism based on morning total testosterone (TT) measured with a validated assay. Most guidelines converge on lower TT limits between 264 and 350 ng/dL, depending on the methodology and population reference standards. The Endocrine Society recommends 264 ng/dL as the cutoff for low TT, whereas the American Urological Association adopts 300 ng/dL, and European and sexual medicine societies (EAU, EAA, ISSM) generally use values around 350 ng/dL (1–5, 15, 16).

In this consensus, TT values <264 ng/dL support the diagnosis of hypogonadism, values >350 ng/dL typically exclude the condition, and men with TT between 264–350 ng/dL require additional biochemical assessment — particularly when SHBG abnormalities are present. In cases of TT between 264 and 350 ng/dL, or conditions that alter SHBG levels, additional laboratory evaluation is required, including measurement of SHBG and albumin to estimate FT using the Vermeulen equation (http://www.issam.ch/freetesto.htm), as the gold-standard method (equilibrium dialysis) for direct FT measurement is expensive and not widely available. In these cases, a cutoff value of 6.5 ng/dL for calculated free testosterone (cFT) is recommended (4, 14, 23, 26).

Once the diagnosis of hypogonadism is confirmed, the next step is to investigate its etiology by distinguishing between primary and secondary causes, which can be done by measuring gonadotropins (LH and FSH) (2–4). Patients with low or inappropriately normal gonadotropin levels should undergo further investigation, including the measurement of prolactin, thyroid function, and transferrin saturation index, to rule out iron overload syndromes (28, 29).

Semen analysis represents a fundamental tool in the evaluation of men with hypogonadism, particularly when fertility preservation is a concern. Beyond its role as the gold standard for assessing male fertility potential, semen analysis provides indirect but clinically relevant information on testicular function, since spermatogenesis is a sensitive marker of the integrity of the HPG axis. Basic parameters, including semen volume, sperm concentration, motility, and morphology, allow characterization of both quantitative and qualitative aspects of spermatogenesis.

In patients with testosterone deficiency, abnormalities such as oligozoospermia, asthenozoospermia, or even azoospermia may reflect impaired Leydig and Sertoli cell function, with consequent reductions in both androgen production and germ cell support (1, 3). Current guidelines emphasize the importance of performing a baseline semen analysis prior to initiating testosterone therapy in men with hypogonadism who desire fertility, as exogenous testosterone suppresses gonadotropin secretion and profoundly impairs spermatogenesis (3, 30). Thus, knowledge of the patient's reproductive potential before therapy is critical for counseling, clinical decision-making, and, when necessary, the institution of fertility-preserving strategies such as cryopreservation or gonadotropin-based treatment (2).

According to the World Health Organization (WHO, 2021), the lower reference limits (5th percentile, p5) and median values (50th percentile, p50) for semen parameters were established based on fertile men whose partners achieved pregnancy within one year of unprotected intercourse. The ejaculate volume presents a lower reference limit of 1.4 mL, with a median of 3.0 mL, when collected after a period of 2–7 days of sexual abstinence. The sperm concentration should be at least 16 million spermatozoa per milliliter, with a median of 66 million/mL, while the total sperm count per ejaculate has a lower reference limit of 39 million and a median value of 210 million spermatozoa. In terms of motility, the total motility (progressive + non-progressive) should reach at least 42%, with a median of 64%, and the progressive motility (PR) alone should be ≥30% (median 55%), which is considered a strong predictor of fertilization capacity. Regarding vitality, at least 54% of spermatozoa must be alive (median 78%), as assessed preferably by eosin-nigrosin staining. For morphology, normal forms should represent ≥4% of the total spermatozoa, with a median of 14%, according to the strict Kruger criteria. The Ph of the semen should be ≥7.2, reflecting adequate function of the accessory glands. Other seminal characteristics include the presence of leukocytes <1 × 10^6^/mL, since higher counts may suggest genital tract inflammation, and liquefaction within 60 minutes, which is considered normal. The viscosity should also be normal, as excessive viscosity can impair sperm motility and hinder fertilization (31).

**Recommendation 05:** The diagnosis of hypogonadism should be confirmed by measurement of TT with a validated assay on fasting morning blood samples obtained on two different days (Class: I, Level of Evidence: C).

**Recommendation 06:** Under conditions that alter SHBG or borderline values of TT, measuring or calculating FT is recommended if clinical suspicion of hypogonadism is strong (Class: I, Level of Evidence: C).

**Recommendation 07:** FSH and LH concentrations should be obtained for distinguishing between primary and secondary causes, with further evaluation to identify the etiology of hypothalamic, pituitary, e/or testicular disfunction (Class: I, Level of Evidence: C).

### Differential diagnosis

Organic hypogonadism results from congenital, structural, or destructive abnormalities that cause permanent impairment of the gonadotropic axis. (4) On the other hand, functional causes lead to the reduction or suppression of gonadotropins and testosterone in a potentially reversible manner, since the underlying condition can be effectively treated (3–5, 32). Table-2 lists some of the common causes of organic and functional hypogonadism.

**Table 2:** Classification of Hypogonadism and Causes of Primary and Secondary Hypogonadism.

ORGANIC	FUNCTIONAL
**Primary Hypogonadism**	
Klinefelter syndrome	Medications (androgen synthesis inhibitors) End-stage renal disease*
Cryptorchidism, anorchia, myotonic dystrophy	
Some types of cancer, chemotherapy, testicular irradiation/damage, orchidectomy	
Orchitis	
Testicular trauma, torsion	
Advanced age
**Secondary Hypogonadism**	
Hypothalamic/pituitary tumor	Hyperprolactinemia
Iron overload syndromes	Anabolic steroid use, glucocorticoids, opioids
Infiltrative/destructive disease of hypothalamus/pituitary	Alcohol and marijuana abuse* Systemic illness*
Idiopathic hypogonadotropic hypogonadism	Nutritional deficiency/excessive Exercise
	Severe obesity, some sleep
	Disorders
	Organ failure (liver, heart,
	and lung) *
	Comorbid illness associated
	with aging*

* Combined primary and secondary hypogonadism but classified to usual predominant hormonal pattern.

Increased prolactin levels, which may result from primary disorders of the hypothalamic-pituitary axis or as a secondary effect of chronic diseases and medications, can lead to suppression of GnRH and gonadotropin synthesis and release (33). Treating the underlying condition associated with hyperprolactinemia does not always resolve hypogonadism. In some cases, hypogonadism linked to pituitary adenomas may not respond to pharmacological treatments and may require more targeted therapy (34).

MOSH is the most common condition associated with functional hypogonadism. (32, 35, 36). Increased estrogen production by adipose tissue, leptin resistance, and chronic inflammation are some of the proposed mechanisms that explain the link between adiposity and male gonadal dysfunction. (35–37) Weight loss, regardless of the method used, can help restore testosterone levels. Failure to recover gonadal function with weight loss may suggest the presence of an underlying organic cause (2, 9, 35, 38–42). In addition, diabetes mellitus represents an important determinant of functional hypogonadism (36). High glucose levels, insulin resistance, and consequent hyperinsulinemia are key contributors to impaired GnRH secretion, ultimately resulting in decreased LH and testosterone levels (9, 36, 43–45).

Anabolic use of androgenic steroids should be considered in the evaluation of functional hypogonadism, as they block hypothalamic GnRH pulsatility and the release of pituitary gonadotropins. The dose and duration of gonadotropic axis blockade affect the likelihood of recovery of gonadal function after cessation. Prolonged use may result in incomplete recovery, even years after discontinuation (46–49).

Relative energy deficiency in sport (RED-S) has recently been used to refer to exercise-associated reproductive health disorders. It results from low-energy diets (intentional or unintentional) and/or excessive exercise, reducing the pulsatile hypothalamic release of GnRH and the release of gonadotropins from the anterior pituitary gland. These changes can promote acute or chronic relative hypogonadism with negative impacts on testosterone levels, fertility, and male bone health (50).

Several other conditions and substances can interfere with the functioning of the gonadotropic axis, leading to male hypogonadism (9). Depression is often associated with sexual dysfunction, and although treatment can alleviate sexual complaints, the use of antidepressants may induce sexual dysfunction. Selective serotonin reuptake inhibitors and serotonin-norepinephrine reuptake inhibitors are drugs associated with a high risk of adverse effects on sex drive and sexual function (51). Opioids, such as morphine, codeine, tramadol, heroin, and oxycodone, can reduce the pulsatility of hypothalamic GnRH and increase prolactin levels by inhibiting dopamine (52, 53). In the absence of other underlying causes of hypogonadism, opioid withdrawal is typically followed by a recovery in serum testosterone levels in a few weeks (52). Long-term use of glucocorticoids can result in androgen deficiency and sperm alterations by suppression of GnRH secretion. Prednisone-equivalent doses as low as 5mg daily can cause hypogonadism in older men (54, 55). It had been demonstrated that men receiving statin therapy present a higher prevalence of biochemical testosterone deficiency compared to nonusers (56). However, despite the mild reduction in circulating testosterone levels observed in this population, the use of statins has not been associated with clinically significant hypogonadal symptoms or relevant impairment in sexual function. Therefore, while statins may slightly decrease serum testosterone concentrations, this effect is not considered to have meaningful clinical repercussions on androgen-related manifestations (56).

**Recommendation 08:** Men with overweight or obesity and Male Obesity Secondary Hypogonadism, lifestyle modifications and weight reduction should be recommended (Class: I, Level of Evidence: A).

**Recommendation 09:** Medications with the potential to suppress testosterone levels—including glucocorticoids, opioids, and anabolic steroids—should be carefully reviewed, and withdrawal or modification should be considered based on the clinical context (Class: II, Level of Evidence: B).

### Role of imaging and complementary exams

Following a thorough clinical and biochemical assessment confirming hypogonadism, identifying the underlying cause is essential.

Klinefelter syndrome is the most common chromosomal cause of primary hypogonadism and is frequently underdiagnosed. Therefore, karyotype analysis in peripheral blood is recommended in men with primary hypogonadism, particularly when testicular volume is reduced (3–5).

Scrotal ultrasound should not be performed routinely, but it can be useful in cases where testicular complaints are present, such as palpable masses, pain, or tenderness (57). It is also valuable for accurately measuring testicular volume in cases of very small testes or when anatomical factors hinder the use of Prader orchidometry, such as in the presence of a large hydrocele or an inguinal testis. Moreover, scrotal ultrasound may play a role in the evaluation of male infertility, particularly for distinguish between obstructive and non-obstructive causes (3, 58) and it is also accepted in situations where physical examination is limited by patient discomfort.

Magnetic resonance imaging (MRI) of the sella turcica, may be indicated in the evaluation of secondary hypogonadism (4, 36, 59). MRI is specifically recommended in cases of suspected organic secondary hypogonadism, as in younger men, when serum testosterone is ≤150 ng/dL (4), in the presence of other pituitary hormone deficiencies, hyperprolactinemia, or symptoms suggestive of a pituitary mass (e.g., new-onset headaches or visual field defects) (3, 4).

Hypogonadism is a recognized risk factor for reduced bone mass and osteoporosis in men. Accordingly, several medical societies recommend dual-energy X-ray absorptiometry (DXA) scanning in men with risk factors such as hypogonadism. Repeat DXA may be considered 1-2 years after initiating testosterone replacement therapy and prior to the use of antiresorptive drug. (3, 4)

Additional evaluations, such as polysomnography, may be warranted based on clinical suspicion of OSA, a condition associated with hypogonadism (3, 4, 60).

**Recommendation 10:** Karyotype should be considered in primary hypogonadism, especially in men with low testicular volume (Class: I, Level of Evidence: B).

**Recommendation 11:** MRI of the sella turcica should be considered in men with suspected organic secondary hypogonadism, particularly in younger individuals with testosterone levels ≤150 ng/dL, coexisting pituitary hormone deficiencies, hyperprolactinemia, or symptoms of pituitary mass effect (Class: I, Level of Evidence: B).

**Recommendation 12:** DXA scanning is recommended for men at increased fracture risk, especially those over 50 years of age with hypogonadism (Class: I, Level of Evidence: B).

## TREATMENT OF MALE HYPOGONADISM

### Formal Indications for Initiating Therapy

It recommends testosterone therapy in men with hypogonadism to induce and maintain secondary sex characteristics and correct symptoms of testosterone deficiency.

Before initiating TRT, contraindications must be systematically assessed, including the presence of prostate or breast cancer, severe untreated obstructive sleep apnea, erythrocytosis (hematocrit >54%), severe heart failure (New York Heart Association class III or IV), and desire for future fertility, as exogenous testosterone suppresses spermatogenesis (3–5). Individualized risk–benefit evaluation and shared decision-making are mandatory. Therapy must aim to restore testosterone levels to the mid-normal physiological range and should be accompanied by regular monitoring of clinical and laboratory parameters.

**Recommendation 13:** Testosterone replacement therapy should be initiated only in men with persistent clinical symptoms of testosterone deficiency and unequivocally low serum testosterone levels, after exclusion of contraindications (Class: I, Level of Evidence: A).

### Therapeutic options available in Brazil

Several testosterone formulations are approved for the treatment of male hypogonadism in Brazil, offering clinicians flexibility to tailor therapy to individual patient preferences, comorbidities, and risk profiles. Available options include injectable testosterone (such as testosterone cypionate, testosterone esters and testosterone undecanoate), and transdermal testosterone (gel). (3–5, 36, 61) Each formulation has distinct pharmacokinetic properties, influencing serum testosterone fluctuations, ease of administration, cost, and side effect profiles.

#### Treatment of Male Hypogonadism: Focus on IM Formulations

Intramuscular (IM) testosterone preparations constitute a cornerstone of TRT in Brazil and globally, owing to their efficacy, relatively low cost, and wide availability. (3, 4)

The long-acting testosterone undecanoate (TU) 1000 mg formulation is currently the preferred IM preparation for sustained TRT. It is administered as a deep intramuscular gluteal injection with an initial loading phase (at weeks 0 and 6), followed by maintenance injections every 10–14 weeks (3, 36). TU provides relatively stable serum testosterone concentrations within the physiological range, with minimal fluctuations between doses, thereby reducing mood and sexual function variability (3–5).

Shorter-acting esters, such as a propionate plus a pool of testosterone esters (250 mg) and cypionate (200 mg), are also widely used in Brazil, particularly in public health settings and in clinical scenarios requiring flexible or temporary TRT (36). These formulations are typically administered every 2 to 3 weeks. However, they produce significant peaks and troughs in serum testosterone levels, which can lead to oscillations in energy, mood, and libido (3). These fluctuations must be discussed with patients prior to initiating therapy.

The route of administration requires deep gluteal injection, which may necessitate trained healthcare personnel or adequate patient education for home administration. Pain or local site reactions may occur but are generally mild and transient (36).

**Recommendation 14:** Intramuscular testosterone formulations should be considered an option for TRT in men with hypogonadism. (Class: I, Level of Evidence: A)

**Recommendation 15:** Long-acting preparations are preferred rather than shorter-acting due to more stable serum levels, reduced fluctuations and lower injection frequency (Class: I, Level of Evidence: A).

#### Treatment of Male Hypogonadism: Focus on Transdermal Testosterone (Gel)

Testosterone gel is a widely used transdermal option in Brazil for treating male hypogonadism, providing a physiological mode of testosterone delivery through skin absorption. It offers advantages of stable serum testosterone concentrations, mimicking normal circadian rhythms without large peaks and troughs typical of injectable formulations (4, 5). Gels (1% or 1,62%) are usually applied once daily on clean, dry, intact skin areas (such as shoulders and upper arms), with absorption occurring over several hours. Steady-state testosterone levels are typically achieved within 2–3 days of consistent use (62).

Transdermal therapy is particularly beneficial for patients desiring a non-invasive, easily adjustable regimen or those at risk of erythrocytosis associated with injectable formulations (2). Nonetheless, clinicians must counsel patients regarding the risk of secondary exposure to others through skin contact, emphasizing the importance of covering the application site and washing hands after application. Dose titration is often necessary, guided by serum testosterone levels obtained 2–4 hours after application and clinical symptom improvement (3). Skin irritation at the application site is a possible side effect but occurs in a minority of patients. The patient should be advised not to shower for up to 2 hours after applying or performing physical activities with excessive sweating, swimming pools or saunas.

Overall, testosterone gel represents an effective and safe alternative for long-term therapy in appropriately selected men with hypogonadism, if patients adhere to application instructions and appropriate monitoring is conducted. That short-acting formulation, particularly transdermal gels, may be preferred at treatment initiation, especially in cases where reversibility or rapid discontinuation may be needed due to adverse effects or diagnostic uncertainty.

**Recommendation 16:** Transdermal testosterone gel is recommended as an effective and safe option for the treatment of male hypogonadism, particularly in patients preferring non-invasive therapy and requiring steady testosterone levels (Class: I, Level of Evidence: A).

#### Other Formulations Available in Brazil

No oral or subcutaneous implants (pellets) testosterone formulations with validated and consistent pharmacokinetics are approved in Brazil for the treatment of male hypogonadism, and they are only available in the compounded market. Subcutaneous testosterone pellets are not recommended by medical specialty societies for the treatment of male hypogonadism in Brazil because compounded formulations do not have robust safety and efficacy studies (1, 63, 64) with high incidence of secondary polycythemia (65).

**Recommendation 17:** Oral testosterone formulations, subcutaneous testosterone pellets (compounded implants and manipulated gel formulations are not recommended for the treatment of male hypogonadism in Brazil, given the absence of approved formulations with validated and consistent pharmacokinetics. (Class: III, Level of Evidence: B)

### Absolute and relative contraindications of testosterone

TRT requires close monitoring due to possible adverse effects, contraindications, and limited long-term safety data. For men who wish to avoid exogenous hormones, are not candidates for TRT, or do not tolerate its effects, non-hormonal pharmacological alternatives are available. (2, 3, 5).

TRT is contraindicated in men with hormone-sensitive tumors such as prostate or breast cancer. Men with prostate abnormalities on physical examination or elevated prostate-specific antigen (PSA) levels should be adequately evaluated before initiating TRT (3). The contraindications and the degrees of recommendation are outlined below.

Identifying and properly managing contraindications is a key step in ensuring the safety of testosterone therapy. While absolute contraindications preclude treatment, relative contraindications require an individualized approach, carefully balancing the potential benefits of TRT against the risks associated with the coexisting condition. Optimal comorbidity management and a rigorous, ongoing monitoring plan are essential when considering TRT in patients with relative contraindications.

#### Absolute Contraindications:

**Male Breast Cancer:** Although rare, these tumors may express hormone receptors, making TRT potentially harmful. Therefore, TRT is contraindicated in patients with a current or past diagnosis of breast cancer (1, 3–5) (Class: I; Level of Evidence: C).

**Active Prostate Cancer:** TRT is contraindicated in the presence of known or suspected prostate cancer without appropriate urological evaluation and treatment. The risk stems from the potential stimulation of androgen-dependent tumors. Guidelines recommend a digital rectal exam and PSA testing before initiating TRT in men aged 55–69 or 40–69 with increased risk (1, 3–5) (Class: I; Level of Evidence: B).

**Desire for Fertility:** Exogenous testosterone suppresses the HPG axis, reducing spermatogenesis and potentially causing infertility. TRT is contraindicated in men who desire short-term fertility. Alternatives such as clomiphene citrate, aromatase inhibitors, or gonadotropins should be considered (1, 3–5) (Class: I; Level of Evidence: B).

#### Relative Contraindications and Precautions:

**Elevated Hematocrit:** Baseline hematocrit > 48% is a relative contraindication to TRT therapy because these men are more likely to develop a hematocrit > 54% when treated with testosterone. The risk of erythrocytosis is somewhat higher with IM formulations, particularly short-acting esters. Men with elevated hematocrit should undergo further evaluation before considering TRT (3, 66) (Class: IIa; Level of Evidence: B).

**Obstructive Sleep Apnea:** The impact of testosterone on OSA remains controversial (3, 60, 67, 68) and routine screening with polysomnography in asymptomatic men under TRT is not recommended. The therapy may worsen sleep apnea, particularly in severe and untreated cases. In patients with mild to moderate, or treated, OSA, TRT may be considered with close monitoring (3, 4, 60) (Class: IIa; Level of Evidence: B).

**Lower Urinary Tract Symptoms (LUTS):** TRT should be used cautiously in patients with severe LUTS (International Prostate Symptom Score (IPSS > 19). Evidence suggests minimal impact on mild to moderate symptoms. Urological evaluation is recommended before initiation, along with ongoing symptom monitoring (1, 3–5) (Class: IIb; Level of Evidence: C).

**Heart Failure:** Testosterone may worsen fluid retention and volume overload, especially in patients with decompensated heart failure. TRT should be avoided or used with extreme caution in patients with decompensation or severe heart failure (NYHA Class III or IV). Although some studies suggest that TRT benefits men with stable heart failure, its safety in decompensated cases has not been established (3, 4, 17) (Class: IIa; Level of Evidence: C).

**Recent Cardiovascular Events:** In men who have experienced acute myocardial infarction (MI) or stroke, TRT should be delayed for at least 6 months to allow clinical stabilization. Although the TRAVERSE trial showed no significant increase in major cardiovascular events with TRT in high-risk men, safety in the immediate post-acute phase was not specifically assessed (3–5, 69) (Class: IIa; Level of Evidence: C).

**Venous Thromboembolism and Thrombophilia:** Despite recent studies not showing a significantly increased risk, caution is advised in patients with a history of venous thromboembolism or known thrombophilic disorders, with individualized assessment (1, 3, 69) (Class: IIb; Level of Evidence: C).

**Previously Treated Prostate Cancer:** TRT may be considered with extreme caution in men previously treated with curative intent and no evidence of recurrence. The decision should be multidisciplinary, with informed consent and rigorous PSA monitoring. (1, 3, 5) (Class: IIb; Level of Evidence: C).

In men with a history of prostate cancer, the decision to initiate testosterone therapy requires individualized, multidisciplinary evaluation. Current evidence suggests that testosterone therapy may be considered in selected men previously treated with curative intent for low-risk disease and with no evidence of recurrence, provided that close urological monitoring is maintained. Clinicians should follow national recommendations from the Brazilian Society of Urology (SBU) and international urological guidelines when evaluating such patients.

**Recommendation 18:** TRT should NOT be initiated in men with:

- Male breast cancer (Class: I, Level of Evidence: C);
- Known or suspected prostate cancer (untreated nodule/induration, PSA > 4 ng/mL or > 3 ng/mL in high-risk individuals without urological evaluation) (Class: I, Level of Evidence: B);
- Active short-term fertility planning (Class: I, Level of Evidence: B).

**Recommendation 19:** Careful evaluation and individualized management are recommended before initiating TRT in men with the following conditions (relative contraindications):

- Baseline hematocrit > 48% or hematocrit at follow-up > 54% (Class: IIa, Level of Evidence: B);
- Untreated severe obstructive sleep apnea (Class: IIa, Level of Evidence: B);
- Decompensated heart failure (NYHA Class III or IV) (Class: IIa, Level of Evidence: C);
- Myocardial infarction or stroke within the past 6 months (Class: IIa, Level of Evidence: C).

**Recommendation 20:** TRT may be considered with caution and close monitoring in men with:

- Severe lower urinary tract symptoms (IPSS > 19), following urological evaluation and treatment optimization (Class: IIb, Level of Evidence: C);
- History of venous thromboembolism or known thrombophilia (Class: IIb, Level of Evidence: C);
- Low-risk prostate cancer treated with curative intent and no evidence of active disease after multidisciplinary discussion and informed consent (Class: IIb, Level of Evidence: C).

### Treatment in men who want to preserve fertility

Testosterone replacement therapy is a well-established treatment for hypogonadism; however, it has been associated with several adverse effects and contraindications reported in the literature, including testicular atrophy and infertility. In this context, gonadotrofins, SERMS and aromatase inhibitors may be used (3, 5, 70).

#### Selective Estrogen Receptor Modulators (SERMs):

Clomiphene citrate (CC), a selective estrogen receptor modulator (SERM), acts by inhibiting estradiol's negative feedback on the HPG axis. This results in increased LH and FSH secretion and subsequent stimulation of Leydig cells, thereby enhancing endogenous testosterone production (71, 72). Even though it is an off-label indication, SERMs has been used to increase sperm count by restoring the physiological function of the HPG axis (3). Studies demonstrate that clomiphene citrate increases testosterone levels in 70–90% of men with secondary hypogonadism with an important role in the treatment of cases of functional hypogonadism and those associated with MOSH and secondary to the use of anabolic steroids. It significantly improves hypogonadal symptoms including sexual function, mood, and energy. It increases sperm count and improves semen parameters in men with infertility related to hormonal abnormalities, and maintains spermatogenesis during treatment, unlike conventional TRT.(3, 5) However, CC is less as effective in the treatment of primary testicular failure (73). Adverse effects include visual disturbances, headache, mood changes, hot flashes, nausea, thrombotic events (rare), and gynecomastia (rare). Contraindications include hypersensitivity to components, severe liver disease, history of thromboembolic events, and primary hypogonadism (73, 74).

**Therapeutic Regimens:** the starting dose is 25 to 50 mg daily, adjusted according to clinical response and testosterone levels. However, in Brazil, only the 50 mg presentation is commercially available. Therefore, the recommendation from this group is to use 50 on alternate days. Titration: Gradually increase the dose up to 50 mg daily based on response. Duration: continuous treatment as long as necessary and well tolerated. There are follow-up studies on CC use for up to 7 years (1, 71, 74).

**Gonadotropins:**

The most used agents are Human Chorionic Gonadotropin (hCG) and LH analog that stimulates Leydig cells to produce testosterone, recombinant FSH that stimulates Sertoli cells and spermatogenesis, and GnRH which is used in pulsatile regimens and stimulates the release of LH and FSH by the pituitary.(5) The main indications for gonadotropin treatment in patients with hypogonadism include promoting normal physical development and maintaining the physical well-being of patients who have experienced tumors, trauma, or malformations (75). Induction of mini puberty in individuals born with congenital hypogonadism is also an indication (76). In addition, since TRT significantly reduces spermatogenesis, gonadotropin treatment is indicated for hypogonadotropic individuals who wish to preserve their fertility (3). Both urinary and recombinant hCG preparations are effective, though recombinant forms offer more consistent dosing and lower immunogenic potential (77, 78). However, it is also important to highlight that HCG or LH analog monotherapies may suppress HPG axis, leading to a decrease in FSH levels and a consequent worsening of semen parameters (3). Adverse effects include injection site reactions, increased risk of fractures, peripheral arterial disease, water retention, cardiac complications, gynecomastia (due to aromatization of testosterone to estradiol), headache, and fatigue. Contraindications include hypersensitivity to components, androgen-dependent tumors, and primary hypogonadism (limited response), thereby emphasizing the need for specialized patient monitoring following treatment (3, 5, 17, 77, 78).

**Therapeutic Regimens:** hCG Monotherapy: Initial dose: 1,500 to 2,000 IU, 2-3 times per week, subcutaneously; dose adjustment based on serum testosterone levels (target: 400–700 ng/dL); duration: at least 3–6 months to assess response (3, 78). Combined Therapy (hCG + FSH): hCG and FSH: 75–150 IU, 2–3 times per week; recommended when there is no adequate response to hCG monotherapy after 6 months or in severe cases of hypogonadotropic hypogonadism (3, 5, 17, 78). Due to the lack of widespread availability of some doses in Brazil, weekly application of 5000 IU hCG is reasonable.

**Aromatase Inhibitors:**

Act by blocking the peripheral conversion of testosterone to estradiol, thereby reducing circulating estradiol levels. Like SERMs, this reduction in estradiol attenuates the negative feedback on the HPG axis, leading to increased LH and FSH and potentially stimulating endogenous testosterone production by the testes. The use of AIs in the treatment of male hypogonadism is off label (3, 5, 71, 79). Indications for use include patients with prolactinomas, obesity, and those who wish to preserve their fertility (3, 80). To assess the toxicity of an aromatase inhibitor, a clinical screening study involving patients with hypogonadism who sought to preserve fertility through treatment with letrozole showed an actual increase in testosterone levels and sperm concentration. Adverse effects include hypertension, increased PSA levels, headaches, reduced bone mineral density, joint and muscle pain, fatigue, mood changes, elevated liver enzymes, increased hematocrit, and elevated LDL cholesterol. Contraindications include hypersensitivity to components, presence of osteoporosis or significant osteopenia, and severe liver disease (81, 82). In this context, follow-up should be conducted to monitor their long-term effects (3).

**Therapeutic Regimens:** Anastrozole: Dose: 0.5–1 mg daily or on alternate days; Duration: continuous treatment as long as necessary and well tolerated (3, 5, 74). Letrozole: Dose: 0.5–2.5 mg daily or weekly; Duration: like anastrozole (74, 81, 82).

#### Gonadotropin releasing hormone agonist (GnRHa) therapy:

Abnormal germ cell development in cryptorchidism results from an endocrinopathy associated with impaired mini puberty, characterized by hormonal imbalance and altered gene expression, rather than a congenital dysgenesis. Low-dose GnRHa therapy in high-risk patients induces a broad transcriptional response within the HPG axis, leading to normal spermatogenesis in 86% of cases and absence of azoospermia. Therefore, post-orchidopexy hormonal treatment is strongly recommended for high-risk cryptorchid boys and may also serve as primary pre-surgical therapy to promote testicular descent and reduce postoperative testicular atrophy (83).

**Recommendation 21:** Men with testosterone deficiency who are interested in fertility should have a reproductive health evaluation performed prior to treatment (Class: I, Level of Evidence: B).

**Recommendation 22:** Use of selective estrogen receptor modulators, human chorionic gonadotropin, aromatase inhibitors, alone or in combination may be offered to selected men with testosterone deficiency desiring to maintain fertility (Class: I, Level of Evidence: C).

### Adjunct and Alternative Therapies: Lifestyle Modifications and Weight Loss

Lifestyle changes, including weight loss and regular physical activity, represent the first-line therapeutic approach for many men with hypogonadism, especially those with MOSH (3–5, 35).

Regular physical exercise, both aerobic and resistance training, can increase testosterone levels, improve body composition, insulin sensitivity, and overall well-being. However, exhaustive and prolonged exercise may lead to a transient decrease in testosterone due to increased cortisol levels. Therefore, moderation and appropriate exercise programming are crucial (84, 85).

Given the lack of evidence, the TRT solely for glycemic control, weight loss, and cardiovascular risk reduction is not recommended (36, 86).

Despite positive preliminary studies, the evidence supporting the use of nutraceuticals and antioxidants in the treatment of male hypogonadism is still considered low, highlighting the need for further clinical trials (1, 3). Therefore, their routine use is not recommended (3, 84).

**Recommendation 23:** Lifestyle changes (weight loss, exercise) should be strongly recommended as first-line therapy for men with overweight and obesity and functional hypogonadism (Class: I, Level of Evidence: A)

**Recommendation 24:** TRT for the sole purposes of glycemic control, weight loss and cardiovascular risk reduction is not recommended (Class: III, Level of Evidence: C).

**Recommendation 25:** Routine supplementation with nutraceuticals or antioxidants is not recommended for the treatment of male hypogonadism (Class: III, Level of Evidence: C)

**Recommendation 26:** Patients considering or using adjuvant and alternative therapies should be informed about the limited evidence base, product variability, and potential risks, and should be appropriately monitored (Class: I, Level of Evidence: C)

### Ethical Conditions for Testosterone Replacement Therapy

In terms of ethical conditions for TRT, it is important to ensure that therapy is administered appropriately, based on clear medical indications, and with full informed consent. Patients must be fully informed about potential benefits, risks (e.g., cardiovascular effects, fertility issues), and the long-term nature of therapy. It is essential to discuss the possibility of side effects like sleep apnea, erythrocytosis (increased red blood cells), and impacts on fertility (3–5, 26). Ethical management involves constant evaluation to decide whether therapy should continue, be adjusted, or stopped based on patient progress.

TRT should not be prescribed for off label uses like general age-related decline in testosterone or for performance enhancement without clear medical justification as it is linked to increased cardiovascular risk and serious complications (87, 88). The use of testosterone in sports for performance enhancement, without medical necessity, is considered doping (89).

## MONITORING OF MALE HYPOGONADISM TREATMENT

### Clinical Monitoring

Clinical monitoring is a fundamental aspect of TRT to ensure therapeutic efficacy, assess symptom resolution, detect adverse effects early, and guide dose adjustments (68, 90). Improvements in symptoms should be evaluated systematically, focusing on domains most affected by testosterone deficiency, such as sexual function (libido, erectile function), mood, vitality, muscle mass, body composition, and quality of life (1–5).

The most consistent and earliest improvements typically occur in sexual desire and libido, often within 3 to 6 weeks of initiating therapy, followed by gradual improvements in erectile function, energy levels, and mood over several months (90). Increases in lean body mass and reductions in fat mass generally become apparent after 12 to 16 weeks of continuous therapy (90). Monitoring should include structured clinical interviews, validated questionnaires when available (such as the International Index of Erectile Function – IIEF), and physical examination focused on body composition and potential adverse effects.

**Figure 2:**
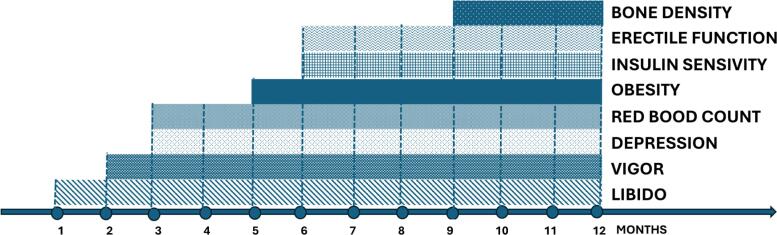
Onset of effects of testosterone replacement therapy as a function of time. Adapted from (16).

A lack of symptomatic improvement despite normalization of serum testosterone levels after an adequate treatment period (typically 6 months) should prompt a reassessment of diagnosis, adherence, comorbidities, and realistic patient expectations (3–5). It is important to counsel patients that improvements vary individually, and that testosterone therapy is not a panacea for all symptoms of aging. A holistic management approach addressing lifestyle, psychological factors, and comorbid illnesses enhances the likelihood of successful outcomes. We emphasize the need to continue treatment even if there is no improvement in symptoms in the initial phase, as there are different timeframes for symptom improvement. Figure-2 summarizes the expected times for symptom improvement after starting TRT.

Timeline of the expected onset of clinical effects after initiating testosterone replacement therapy, including sexual, metabolic, psychological, and musculoskeletal domains.

While the goal of therapy is clinical improvement and normalization of testosterone within the mid-normal range, a target value between 450–600 ng/dL is considered appropriate, aligning with the European Association of Urology (EAU) and Endocrine Society recommendation (3, 4).

**Recommendation 27:** During testosterone replacement therapy, clinicians should perform regular clinical evaluations to assess symptomatic improvement in libido, sexual function, vitality, mood, and body composition, using structured interviews and validated tools where appropriate (Class: I, Level of Evidence: A)

### Dose Adjustments and Changes in Therapy Strategies

The need to change the therapeutic strategy may arise due to various factors, including inadequate symptomatic or laboratory response at the maximum tolerated dose of the current formulation, intolerable or persistent adverse effects, patient preference for a different route or dosing frequency, or cost and availability issues (5, 17). Management includes dose reduction, increased interval between injections, switching to a transdermal formulation, or performing therapeutic phlebotomy (68). If it persists, discontinuation should be considered (Table-3).

**Table 3:** Dose adjustments and changes in therapeutic strategies. Adapted from (1, 3).

Clinical Situation	Recommended Action	Reevaluation Time
Start of TRT	Start with the standard dose of the chosen formulation	-
Testosterone levels within target range (400–700 ng/dL)	Maintain current dose	Monitor annually
Testosterone levels below target range and symptoms persist	Gradually increase dose	Reevaluate in 6–12 weeks
T levels above target	Gradually reduce dose	Reevaluate in 6–12 weeks
Dose-related adverse effects (e.g., mild erythrocytosis)	Gradually reduce dose	Reevaluate in 6–12 weeks
Symptoms persist despite testosterone levels within target range	Reassess diagnosis, consider other causes, discuss discontinuation	Individualized decision

Monitoring testosterone levels may vary depending on the pharmacokinetics of each formulation. When using short-acting options, it should be considered that the maximum plasma peak occurs rapidly around the first 2-5 days, with a median nadir around 15-20 days. This allows doses to be administered at intervals ranging from 2 to 4 weeks, depending on the patient's clinical response. The long-acting formulation tends not to peak, maintaining testosterone levels close to physiological levels for a period of 10 to 14 weeks. Testosterone measurement is recommended at the end of the ampoule administration interval. In the transdermal gel option, serum testosterone should be evaluated 2 to 8 hours after gel application, after the patient has been in treatment for at least 1 week. It is recommended to evaluate serum testosterone levels 2 to 3 months after the start of treatment (3, 4).

Transitioning to other formulations should be done in a way that minimizes fluctuations in testosterone levels and symptoms. Temporary or permanent discontinuation is indicated in the absence of clinical benefit after 3–6 months of adequate testosterone levels, development of contraindications (e.g., prostate cancer), severe unmanageable adverse effects, or patient desire (3).

Reassessment of symptoms, hormonal levels, and comorbidities should be performed after discontinuation, as the HPG axis may take weeks to months to recover. In selected cases, alternative therapies may be implemented. (4, 91). The algorithm for dose adjustments and changes in therapeutic strategies is shown in Table-1.

**Recommendation 28:** Serum testosterone levels should be monitored at 3, 6, and 12 months after starting TRT, and every 6–12 months thereafter, with dose adjustments to achieve levels within the mid-tertile reference range (Class: I, Level of Evidence: A)

**Recommendation 29:** Transitioning between different testosterone formulations is a valid strategy in cases of inadequate response, adverse effects, or patient preference (Class: I, Level of Evidence: C)

### Monitoring and management of side effects

Testosterone dosage should be adjusted to achieve serum levels within the mid-tertile reference range. If no clinical benefit is observed or significant changes in hematocrit or PSA levels occur after 6–12 months of therapy, TRT should be adjusted or discontinued. In such cases, the hormonal axis and comorbidities must be reassessed, and alternative therapeutic options considered (90).

TRT is associated with several potential adverse effects, including gynecomastia, acne, increased skin oiliness, baldness, erythrocytosis, reduced sperm production (potentially leading to infertility), decreased testicular volume, fluid retention and/or edema, symptoms related to obstructive sleep apnea and hypoventilation syndrome and growth of androgen-dependent neoplasms (68). Local irritation is common, and rotating application sites and moisturizing the skin may help. If persistent, consider changing the formulation (1, 3–5).

Gynecomastia has been observed in men receiving TRT when elevated estrogen levels occur. Routine monitoring of estradiol is not recommended. Tamoxifen may also be used to reduce breast tissue enlargement. In symptomatic cases that persist for at least 12 months despite medical management, elective plastic surgery may be considered (92–94).

Acne is a common side effect of TRT due to increased sebaceous gland activity. Topical therapies such as retinoids, benzoyl peroxide, azelaic acid, and/or combinations of topical agents are first-line treatments in managing mild to moderate acne. In more severe cases, oral antibiotics or isotretinoin may be indicated. Educating patients on proper skin care is also essential to help minimize acne outbreaks. Referral to a dermatologist should be considered for patients with persistent or severe acne (68, 95).

Patients should be assessed for testosterone and hematocrit levels at 3, 6 and 12 months after starting TRT, with subsequent annual evaluations (3–5, 15, 26). Elevation of hematocrit above the limit of normal is the most common adverse effect of TRT and has been associated with cardiovascular events and/or venous thromboembolism (VTE) due to blood hyperviscosity (3, 66, 68, 96). If the hematocrit level exceeds 54% during treatment, it is advisable to temporarily suspend therapy until the levels return to a safe range(68). Once normalized, TRT can be resumed at lower doses, potentially with a different formulation. The testosterone gel, for example, tends to have a lesser impact on hematocrit levels. The decision to perform phlebotomy should be made on a case-by-case basis. Additionally, addressing factors such as obesity, sleep apnea, and smoking may help reduce the risk of erythrocytosis (3, 4, 26). If a VTE episode occurs while in TRT and hematocrit values are within normal range, an underlying undiagnosed thrombophilia can be the cause (3, 5, 97). Lipid and glycemic profile should be evaluated at baseline and annually during testosterone therapy (3).

TRT is not recommended for men seeking fertility, as exogenous testosterone suppresses the HPT axis and subsequently impairs spermatogenesis (1–5, 26). The extent of the negative impact of TRT on fertility is variable. Evidence from studies in healthy men suggests that baseline fertility is typically restored in most patients within 24 months following cessation of TRT. However, data regarding this recovery in infertile males remain limited (1). Depending on the underlying cause of hypogonadism, and after evaluating the individual's fertility, it may be advisable to consider alternative medications to TRT (1–5). Every patient should be inquired about their future fertility plans regardless of age and should be counseled about fertility preservation options. For those with fertility concerns, an assessment should be conducted through semen analysis and partner's fertility status (3). Referral to fertility specialists should be considered.

There is an increased risk of fluid retention and edema, which may exacerbate pre-existing edematous conditions, such as heart failure (4, 98). TRT does not appear to worsen LUTS in men who do not have severe LUTS prior to treatment. The effects of testosterone on men with severe LUTS remain unclear, as this group was excluded from clinical studies (3, 99–101). TRT in men with obstructive sleep apnea may exacerbate symptoms, so caution is advised when initiating TRT in these patients (60, 102).

The risk of prostate cancer should be evaluated before starting TRT using PSA measurements and digital rectal examination in men after 45 years and especially those at higher risk for prostate cancer (men of African descent and those with a first-degree relative with diagnosed prostate cancer or previously positive prostate biopsy, and in those with baseline PSA concentrations >1ng/mL at age 40 years or > 2ng/mL at age 60 years) (103). Recommendation to reassess PSA measurements again 3, 6 and 12 months after initiation (3, 5). After the first 12 months, local guidelines for prostate cancer screening for the general population should be followed which emphasize shared decision-making and risk stratification rather than universal testing (5). A urological evaluation should be requested prior or during TRT if any abnormalities are detected during the digital rectal examination, if there is a new onset or worsening of LUTS (5, 30). It is important to emphasize that a modest initial rise in PSA may occur within the first 3 to 6 months of TRT (up to 1.0 ng/dL), reflecting increased prostatic androgenic stimulation; however, PSA levels generally stabilize thereafter (104, 105). Persistent increases beyond the first year, a velocity of rise exceeding 0.75 ng/mL/year, or a rise >1.4 ng/mL within the first 12 months should prompt referral to a urologist (1, 3–5). PSA thresholds should be interpreted in an age-specific manner: values >2.5 ng/mL in men aged 40–49 years, >3.5 ng/mL in men aged 50–59 years, and >4.0 ng/mL in men aged ≥60 years are considered abnormal and warrant further evaluation (103).

Although not a common side effect of TRT, men with hypogonadism should be screened for osteoporosis (3, 4). Recently, the TRAVERSE study reported a higher incidence of fractures in men receiving TRT (106). However, this increase was observed mainly during the initial months of treatment and included all types of fractures. When considering only low-impact fractures, the risk was similar between the TRT and placebo groups (106). Although previous studies have shown improvements in BMD with TRT, it should not be used as a standalone treatment for patients at high risk of fracture, as evidence supporting its anti-fracture efficacy remains limited (4, 5, 26, 106).

**Recommendation 30:** Testosterone and hematocrit levels should be measured at 3, 6, and 12 months after the initiation of TRT, and then annually. If hematocrit exceeds 54%, TRT should be discontinued until levels decrease to a safe range. Treatment can then be resumed with a lower dose and a transdermal formulation (Class: I, Level of Evidence: B).

**Recommendation 31:** A digital rectal examination and PSA test should be performed at 3 and 12 months after starting TRT, and subsequently according to local guidelines for prostate cancer screening in the general population (Class: II, Level of Evidence: C).

**Recommendation 32:** Further evaluation should be considered if there is an increase in serum PSA levels > 1.4 ng/mL within 12 months of TRT, a confirmed PSA > 4 ng/mL at any time, a detection of a prostatic abnormality on digital rectal examination, or a significant worsening of LUTS (Class: II, Level of Evidence: C)

### Discontinuation and Reassessment of Therapeutic Need

Organic male hypogonadism is generally considered a lifelong condition. However, spontaneous reversal may occur in select cases. Studies on congenital secondary hypogonadism have reported reversal in approximately 10-15% of patients undergoing TRT (107, 108). Clinicians should remain alert to this possibility, particularly in the presence of unexpected testicular enlargement during treatment (109, 110). A recent study involving men with congenital hypogonadotropic hypogonadism identified several predictors of reversibility, including larger testicular volume, absence of micropenis, and higher serum FSH levels. These features are consistent with Pasqualini syndrome (fertile eunuch phenotype). In contrast, the presence of pathogenic ANOS1 mutations was associated with irreversible hypogonadism (111).

While reversal is rare in cases of organic hypogonadism, functional hypogonadism may be resolved with the treatment of underlying conditions (2). Potentially reversible causes include hyperprolactinemia, end-stage renal disease, opioid use, previous anabolic steroid use, glucocorticoid therapy, excessive alcohol or cannabis use, systemic illness, nutritional deficiencies or excessive exercise, obesity, metabolic syndrome, certain sleep disorders, and other chronic diseases (4, 5, 36). When a reversible cause is identified, a trial discontinuation of TRT should be considered to reassess HPG axis function. The timing of this reassessment should account for the pharmacokinetics of the testosterone preparation used and the clinical scenario, including symptom resolution and biochemical normalization of testosterone levels. If endogenous testosterone levels remain normal and symptoms resolve, tapering or discontinuation of TRT may be appropriate, with close monitoring for recurrence of hypogonadal symptoms or hormonal decline (2–5).

## CONCLUSIONS

Male hypogonadism is a multifaceted condition that requires timely recognition, careful diagnostic evaluation, and individualized management strategies. Incorporating evidence-based therapies, such as testosterone replacement therapy and selective estrogen receptor modulators, in the context of multidisciplinary care, is essential to optimize clinical outcomes and address patient-specific goals, including potential fertility preservation.

This position paper underscores the importance of continuous education, adherence to updated guidelines, and collaborative care across specialties. By providing practical recommendations and highlighting emerging therapeutic options, it seeks to enhance clinician awareness, reduce underdiagnosis, and improve the quality of care for men affected by hypogonadism.

## Data Availability

uninformed
